# Success and Challenges Querying OMOP-transformed EHR Data from Different Healthcare Organizations

**DOI:** 10.1055/a-2668-3461

**Published:** 2025-08-26

**Authors:** Colin T. Purmal, Michael E. Matheny, Lucila Ohno-Machado, Gary Tarasovsky, Rick Larsen, Mary A. Whooley

**Affiliations:** 1 San Francisco VA Health Care System, San Francisco, California, United States; 2 University of California, San Francisco, California, United States; 3 VA Tennessee Valley Health Care System, Nashville, Tennessee, United States; 4 Vanderbilt University, Nashville, Tennessee, United States; 5 Department of Biomedical Informatics and Data Science, Yale School of Medicine, New Haven, Connecticut, United States

**Keywords:** interoperability, common data model, health information exchange

## Abstract

**Background:**

Adoption of a common data model (CDM), such as the Observational Medical Outcomes Partnership (OMOP) CDM, is a critical component of health information exchange, but the extent to which CDMs facilitate patient-level interoperability is unclear. We sought to determine the feasibility of using a CDM for health information exchange between two healthcare organizations.

**Materials and Methods:**

We executed a single Structured Query Language (SQL) query on OMOP-transformed data from the University of California, San Francisco (UCSF) and the San Francisco VA Healthcare System for five patients.

**Results:**

The SQL query was successfully executed, and complementary healthcare information was obtained for five of five patients, but interoperability was limited by (1) lack of uniform patient identifiers, (2) use of different coding vocabularies, and (3) variations in mapping of source data to the CDM.

**Conclusion:**

Although transformation of EHR data to a CDM can facilitate health information exchange, our study suggests that patient-level interoperability of a CDM may require further alignment of both semantics and syntax.

## Introduction

United States (US) healthcare encompasses a diverse array of provider and payer organizations that rely on a variety of electronic health record (EHR) systems which collect and store data in different manners. The wide range of data capture, coding, organization, and storage methods makes information sharing difficult and leads to care fragmentation when patients inevitably obtain services in different medical settings. Recognizing these challenges, the US 21st Century Cures Act included provisions to promote EHR data interoperability, prevent information blocking, and ensure flow of crucial healthcare data to end-user clinicians. 1

To implement this legislation, the Office of the National Coordinator (ONC) created “The Trusted Exchange Framework and Common Agreement” that describes a set of non-binding, foundational principles for trust policies and practices to facilitate exchange among health information networks (HINs). 2 The ONC also created the United States Core Data for Interoperability, a set of individual data elements that have been deemed critically important to promote interoperability. 3 Capture of these data elements is one of the criteria required for certification of application programming interfaces for patient and population services. 3

However, capture of data elements is only the first step required for health information exchange. Structured data must also be accessible, retrievable, and readable by providers at other healthcare organizations. Network services, such as Carequality (used in Epic's Care Everywhere) and CommonWell Health Alliance, facilitate health information exchange, but both structured and unstructured data elements are presented in cumbersome text summaries that preclude easy retrieval by providers at other healthcare organizations. To find a laboratory value, for example, a clinician must search through a lengthy healthcare summary (including patient demographics, medications, procedures, immunizations, and vital signs) rather than viewing the result in a structured, labeled, and searchable data field as it would be seen in the source EHR.

The Fast Healthcare Interoperability Resources (FHIR) standard enables secure exchange of health information through an application programming interface. It can be used in a wide range of settings and with different health information systems, but it does not generate humanly readable output. Of note, FHIR focuses on rapid, flexible transfer of healthcare data and does not require harmonization of source data. This difference sets FHIR apart from common data models (CDMs) such as the Observational Medical Outcomes Partnership (OMOP) CDM, differentiating it as a separate tool for interoperability. Ongoing efforts, like OMOP on FHIR, have used both tools to share harmonized data between organizations. 4

Although the USCDI sets a foundation for sharing of EHR information to support patient care, it does not stipulate any specific CDM, enforce universal standards or taxonomies for the collection of variables, specify acceptable values, or establish internal relationships between data elements. CDMs provide valuable frameworks, structure, methods, and tools to convert source data into a common language (even allowing conversion of records in written languages other than English from healthcare systems beyond the US such as France 5 ) that can be spoken and understood by different healthcare systems. Therefore, use of a CDM, such as the Biomedical Research Integrated Domain Group (BRIDG) 6 or harmonize, 7 could theoretically improve interoperability and health information exchange, depending on source data structure.

OMOP was a public–private partnership that was initially organized in response to the FDA amendments Act of 2007 due to the need for greater pharmacovigilance (The Future of Drug Safety). 8 Because pharmacovigilance often requires observational data generated outside of large randomized controlled trials, OMOP developed a person-centric relational data model called the OMOP-CDM. 8 9 10 The OMOP-CDM featured multiple domains including demographics, observation periods, drug exposures, condition occurrences, procedures, visits, and clinical observations. 9 10 Further expansion and curation of the OMOP-CDM and the creation of complementary analytical tools has been overseen by the not-for-profit Observational Health Data Sciences and Informatics (OHDSI) collaborative. 11

**Fig. 1:**
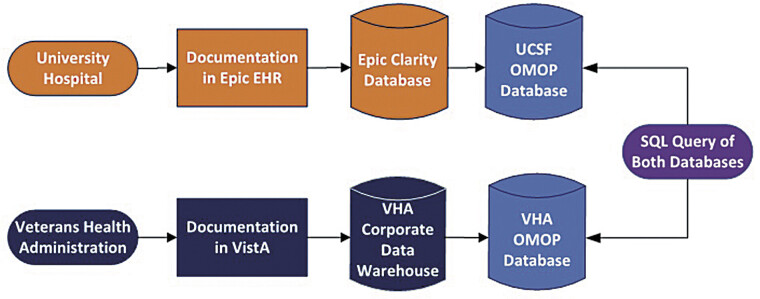
Information flow diagram for the University of California, San Francisco (UCSF), and the Veterans Health Administration (VHA) Observational Medical Outcomes Partnership (OMOP)-transformed electronic health records. EHR, electronic health record; SQL, Structured Query Language; VistA, Veterans Health Information Systems and Technology Architecture.

Multiple publications document the conversion of a wide variety of source data to the OMOP-CDM and successful analysis of these data using interoperable common methods that are source agnostic. 9 12 13 14 15 These studies demonstrate a key feature of the OMOP-CDM—healthcare data interoperability for research—but have not examined its potential use for clinical and operational healthcare coordination. Like most healthcare CDMs, OMOP stores data in star schema relational database management systems accessed using Structured Query Language (SQL), but whether conversion of source data to the OMOP-CDM enables a single SQL query to obtain complementary clinical data across different healthcare systems is unknown. Therefore, we sought to query OMOP-transformed data from two healthcare systems using a single SQL script designed to obtain healthcare information for patients seen at both the institutions Fig. 1 .

## Materials and Methods

As part of the patient-centered Scalable National Network for Effectiveness Research (pSCANNER) project, 16 health data from the San Francisco VA (SFVA) and University of California, San Francisco (UCSF) were extracted, transformed, and loaded (ETL) into parallel databases using the OMOP-CDM. Data from the SFVA was extracted from the Corporate Data Warehouse, a relational database that contains a nightly ETL of clinical data from the Veterans Health Information Systems and Technology Architecture (VistA). 17 Data from UCSF's Epic Clarity Data Model were extracted and transformed to the OMOP-CDM. 16

**figure 2:**
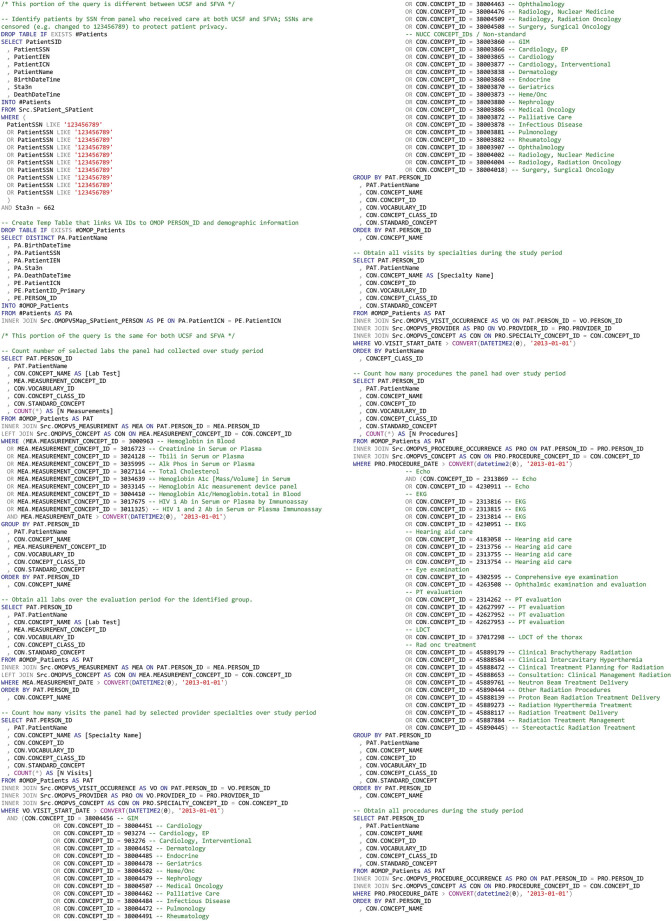
Query Language code used for the study.

After obtaining study approval from the UCSF Institutional Review Board and the SFVA Research & Development Committee, we identified nine deceased patients who were known to have received care at both the institutions. Each patient was identified using Social Security Number (SSN), and we used SSN/OMOP PERSON_ID crosswalks to identify OMOP PERSON_ID at both VA and UCSF. All nine patients had records within VHA, but only five out of nine patients were identified within UCSF OMOP. After identification of patients' OMOP PERSON_IDs, both the VHA and UCSF OMOP instances were queried using the SQL code shown in Fig. 2 . The SQL query was designed to output counts of laboratory tests, clinical visits, and procedures by joining five (Concept, Person, Procedure_occurrence, Provider, and Visit_occurrence) OMOP tables. The OMOP Concept IDs used to obtain count data are shown in Table 1 . Initially only VHA specific non-standard CONCEPT_IDs (based on the National Uniform Claim Committee [NUCC] vocabulary) were used for physician specialty, but after finding no results at UCSF, the query was expanded to include standard CONCEPT_IDs (based on Medicare Specialty) which identified visit occurrences at UCSF. Minor changes in the code were required between the VHA and UCSF OMOP instance queries due to different schema and project provisioning naming conventions. Output from the queries was collected and organized in tabular format.

**Table 1:** Concept IDs used to query Observational Medical Outcomes Partnership (OMOP)-transformed electronic health record data from two different healthcare systems

Concept name (Truncated)	OMOP domain	OMOP concept class	Vocabulary source	Standard concept	CONCEPT_ID
Alkaline phosphatase	MEASUREMENT	Laboratory test	LOINC	Standard	3035995
Bilirubin, Total	MEASUREMENT	Laboratory test	LOINC	Standard	3024128
Cholesterol	MEASUREMENT	Laboratory test	LOINC	Standard	3027114
Creatinine	MEASUREMENT	Laboratory test	LOINC	Standard	3016723
Hemoglobin	MEASUREMENT	Laboratory test	LOINC	Standard	3000963
Hemoglobin A1c	MEASUREMENT	Laboratory test	LOINC	Standard	3034639
Hemoglobin A1c	MEASUREMENT	Clinical observation	LOINC	Standard	3033145
Hemoglobin A1c	MEASUREMENT	Laboratory test	LOINC	Standard	3004410
HIV 1 Ab	MEASUREMENT	Laboratory test	LOINC	Standard	3017675
HIV 1 & 2 Ab + A47	MEASUREMENT	Laboratory test	LOINC	Standard	3011325
Cardiology	Provider	Physician specialty	Medicare specialty	Standard	38004451
Clinical cardiac electrophysiology	Provider	Physician Specialty	Medicare Specialty	Standard	903274
Dermatology	Provider	Physician specialty	Medicare specialty	Standard	38004452
Endocrinology	Provider	Physician specialty	Medicare specialty	Standard	38004485
Geriatric medicine	Provider	Physician specialty	Medicare specialty	Standard	38004478
Hematology/Oncology	Provider	Physician specialty	Medicare specialty	Standard	38004502
Hospice and palliative care	Provider	Physician specialty	Medicare specialty	Standard	38004462
Infectious disease	Provider	Physician specialty	Medicare specialty	Standard	38004484
Internal medicine	Provider	Physician specialty	Medicare specialty	Standard	38004456
Interventional Cardiology	Provider	Physician Specialty	Medicare Specialty	Standard	903276
Medical oncology	Provider	Physician specialty	Medicare specialty	Standard	38004507
Nephrology	Provider	Physician specialty	Medicare specialty	Standard	38004479
Nuclear medicine	Provider	Physician specialty	Medicare specialty	Standard	38004476
Ophthalmology	Provider	Physician specialty	Medicare specialty	Standard	38004463
Pulmonary disease	Provider	Physician specialty	Medicare specialty	Standard	38004472
Radiation oncology	Provider	Physician specialty	Medicare specialty	Standard	38004509
Rheumatology	Provider	Physician specialty	Medicare specialty	Standard	38004491
Surgical oncology	Provider	Physician specialty	Medicare specialty	Standard	38004508
Allopathic & osteopathic physicians, dermatology	Provider	Physician specialty	NUCC	Non-standard	38003838
“,” IM, Cardiovascular disease	Provider	Physician specialty	NUCC	Non-standard	38003865
“,” IM, Clinical cardiac electrophysiology	Provider	Physician specialty	NUCC	Non-standard	38003866
“,” IM, Endocrinology, diabetes & metabolism	Provider	Physician specialty	NUCC	Non-standard	38003868
“,” IM, Geriatric medicine	Provider	Physician specialty	NUCC	Non-standard	38003870
“,” IM, Hematology & oncology	Provider	Physician specialty	NUCC	Non-standard	38003873
“,” IM, Hospice and palliative medicine	Provider	Physician specialty	NUCC	Non-standard	38003872
“,” IM, Infectious disease	Provider	Physician specialty	NUCC	Non-standard	38003878
“,” IM, Interventional cardiology	Provider	Physician specialty	NUCC	Non-standard	38003877
“,” IM, Medical oncology	Provider	Physician specialty	NUCC	Non-standard	38003886
“,” IM, Nephrology	Provider	Physician specialty	NUCC	Non-standard	38003880
“,” IM, Pulmonary disease	Provider	Physician specialty	NUCC	Non-standard	38003881
“,” IM, Rheumatology	Provider	Physician specialty	NUCC	Non-standard	38003882
“,” Ophthalmology	Provider	Physician specialty	NUCC	Non-standard	38003907
“,” Radiology, Nuclear radiology	Provider	Physician specialty	NUCC	Non-standard	38004002
“,” Radiology, Radiation oncology	Provider	Physician specialty	NUCC	Non-standard	38004004
“,” Surgery, Surgical oncology	Provider	Physician specialty	NUCC	Non-standard	38004018
Clinical brachytherapy	Procedure	CPT4 hierarchy	CPT4	Classification	45889179
Clinical intracavitary hyperthermia	Procedure	CPT4 hierarchy	CPT4	Classification	45888584
Clinical treatment planning for radiation treatment	Procedure	CPT4 hierarchy	CPT4	Classification	45888472
Comprehensive eye examination	Procedure	Procedure	SNOMED	Standard	4302595
Consultation: Clinical management for radiation treatment	Procedure	CPT4 hierarchy	CPT4	Classification	45888653
Echocardiography	Procedure	Procedure	SNOMED	Standard	4230911
Echocardiography, Transthoracic, 2D	Procedure	CPT4	CPT4	Standard	2313869
ECG with at least 12 leads; interpretation and report only	Procedure	CPT4	CPT4	Standard	2313816
ECG with at least 12 leads; tracing only	Procedure	CPT4	CPT4	Standard	2313815
ECG with at least 12 leads; with interpretation and report	Procedure	CPT4	CPT4	Standard	2313814
Fitting of hearing aid	Procedure	Procedure	SNOMED	Standard	4183058
Hearing aid check; binaural	Procedure	CPT4	CPT4	Standard	2313756
Hearing aid check; monaural	Procedure	CPT4	CPT4	Standard	2313755
Hearing aid examination and selection; binaural	Procedure	CPT4	CPT4	Standard	2313754
Hyperthermia	Procedure	CPT4 hierarchy	CPT4	Classification	45889273
Low-dose computed tomography of thorax	Procedure	Procedure	SNOMED	Standard	37017298
Neutron beam treatment delivery	Procedure	CPT4 hierarchy	CPT4	Classification	45889761
Ophthalmic examination and evaluation	Procedure	Procedure	SNOMED	Standard	4263508
Other radiation procedures	Procedure	CPT4 hierarchy	CPT4	Classification	45890444
Physical therapy evaluation (Deprecated)	Procedure	CPT4	CPT4	Standard	2314262
Physical therapy evaluation, high complexity	Procedure	CPT4	CPT4	Standard	42627997
Physical therapy evaluation, low complexity	Procedure	CPT4	CPT4	Standard	42627952
Physical therapy evaluation, moderate complexity	Procedure	CPT4	CPT4	Standard	42627953
Proton beam treatment delivery	Procedure	CPT4 hierarchy	CPT4	Classification	45888139
Radiation treatment delivery	Procedure	CPT4 hierarchy	CPT4	Classification	45888117
Radiation treatment management	Procedure	CPT4 hierarchy	CPT4	Classification	45887884
Stereotactic radiation treatment delivery	Procedure	CPT4 hierarchy	CPT4	Classification	45890445
Stretcher	Observation	Physical object	SNOMED	Non-standard	4230951

## Results

Table 2 presents the output of count values from the SQL query. Each row shows the count of a specific test, visit occurrence, or procedure occurrence within each facility. Columns represent the source of these data counts (SFVA, UCSF, or the combined sum of counts across both healthcare systems). The most commonly occurring measurement was creatinine, with a total of 187 separate measurements (159 VHA + 28 UCSF) across 5 patients. The most commonly occurring procedure was EKG interpretation, with a total report of 82 EKG interpretations (61 VHA + 21 UCSF). As above, UCSF and SFVA used separate concepts to describe visit occurrence (UCSF uses Medicare Specialty whereas VHA uses NUCC). At UCSF, the most common visit occurrence was with Ophthalmology (22 visits total), whereas at the SFVA the most common occurrence was with General Internal Medicine (231 visits total).

**Table 2:** Counts of laboratory tests, specialist visits, and procedures obtained based on CONCEPT IDs in Table 1

OMOP CONCEPT	Vocab	Patient 1			Patient 2			Patient 3			Patient 4			Patient 5			Sum		
		UC	VA	C	UC	VA	C	UC	VA	C	UC	VA	C	UC	VA	C	UC	VA	C
Measurement Table																			
Alkaline Phosphatase	LOINC	14	0	14	3	5	8	29		29	13	1	14	10	3	13	69	9	78
Bilirubin	LOINC	14		14	3	5	8	29		29	13	1	14	10	2	12	69	8	77
Cholesterol	LOINC	1		1	4		4	18		18	5	1	6	5	4	9	33	5	38
Creatinine	LOINC	55		55	6	6	12	63		63	29	2	31	6	20	26	159	28	187
Hemoglobin	LOINC	51		51	5	3	8	42		42	26	2	28	8	19	27	132	24	156
Hemoglobin A1c in Serum or Plasma	LOINC			0			0			0			0			0	0	0	0
Hemoglobin A1c measurement device panel	LOINC			0			0			0			0			0	0	0	0
Hemoglobin A1c/Hemoglobin total in Blood	LOINC	3		3			0			0	3		3	1		1	7	0	7
HIV 1 + 2 AB [Presence] In Serum	LOINC	1		1			0			0	2		2	1		1	4	0	4
HIV 1 Ab in Serum or Plasma by Immunoassay	LOINC			0			0			0			0			0	0	0	0
Procedure Table																			
Comprehensive Eye Examination	SNOMED	1		1	1		1	21		21	2		2	1		1	26	0	26
Echocardiography	SNOMED	1		1			0	1		1	1		1	3		3	6	0	6
Echocardiography Transthoracic, Real-time	CPT4	1		1		2	2			0	15	2	17	7	12	19	23	16	39
Electrocardiogram, interpretation and report only	CPT4	24		24		5	5	16		16	15	1	16	6	15	21	61	21	82
Electrocardiogram, tracing only	CPT4	24		24		3	3	16		16			0	1	16	17	41	19	60
Electrocardiogram with interpretation and report	CPT4			0			0			0			0			0	0	0	0
Fitting of Hearing Aid	CPT4			0			0			0			0	1		1	1	0	1
Hearing Aid Check; Binaural	CPT4			0			0	1		1	11		11	2		2	14	0	14
Hearing Aid Check; Monaural	CPT4			0			0			0	4		4			0	4	0	4
Hearing Aid Examination and Selection; Binaural	CPT4			0			0			0			0			0	0	0	0
Low Dose Computed Tomography of Thorax	SNOMED			0			0			0	2		2			0	2	0	2
Ophthalmic Examination and Evaluation	SNOMED			0			0	10		10	24		24			0	34	0	34
Physical Therapy Evaluation (Deprecated)	CPT4	2		2			0	3		3	1		1	1	1	2	7	1	8
Physical Therapy Evaluation; High Complexity	CPT4			0			0	1		1	1		1			0	2	0	2
Physical Therapy Evaluation; Low Complexity	CPT4			0			0			0	1		1		1	1	1	1	2
Physical Therapy Evaluation; Moderate Complexity	CPT4			0			0			0	1		1			0	1	0	1
Provider Visit Occurrence Table																			
General Internal Medicine	NUCC	33		33	17		17	95		95	51		51	35		35	231	0	231
Cardiology	NUCC	27		27			0	18		18	14		14	11		11	70	0	70
Cardiology, Electrophysiology	NUCC			0			0	1		1	2		2			0	3	0	3
Cardiology, Interventional	NUCC	2		2			0	8		8	9		9	1		1	20	0	20
Dermatology	NUCC			0			0	6		6			0			0	6	0	6
Endocrinology	NUCC	1		1			0	1		1			0			0	2	0	2
Geriatrics	NUCC			0			0	2		2	2		2	1		1	5	0	5
Hematology / Oncology	NUCC			0			0	18		18			0			0	18	0	18
Nephrology	NUCC	5		5			0			0			0			0	5	0	5
Medical Oncology	NUCC			0			0	2		2	6		6			0	8	0	8
Palliative Care	NUCC			0			0	4		4			0			0	4	0	4
Infectious Disease	NUCC			0			0			0	9		9			0	9	0	9
Pulmonology	NUCC	1		1			0			0			0	8		8	9	0	9
Rheumatology	NUCC	2		2			0	3		3			0			0	5	0	5
Ophthalmology	NUCC			0			0	32		32	26		26			0	58	0	58
Radiology, Nuclear Medicine	NUCC			0			0	1		1	3		3	1		1	5	0	5
Radiology, Radiation Oncology	NUCC			0			0			0			0			0	0	0	0
Surgery, Surgical Oncology	NUCC			0			0			0			0			0	0	0	0
General Internal Medicine	Medicare Specialty			0			0			0			0		2	2	0	2	2
Cardiology	Medicare Specialty			0			0			0			0		16	16	0	16	16
Cardiology, Electrophysiology	Medicare Specialty			0			0			0			0			0	0	0	0
Cardiology, Interventional	Medicare Specialty			0			0			0			0		7	7	0	7	7
Dermatology	Medicare Specialty			0			0			0			0			0	0	0	0
Endocrinology	Medicare Specialty			0			0			0			0			0	0	0	0
Geriatrics	Medicare Specialty			0			0			0			0		4	4	0	4	4
Hematology / Oncology	Medicare Specialty			0			0			0			0			0	0	0	0
Nephrology	Medicare Specialty			0			0			0			0			0	0	0	0
Medical Oncology	Medicare Specialty			0			0			0			0			0	0	0	0
Palliative Care	Medicare Specialty			0			0			0			0			0	0	0	0
Infectious Disease	Medicare Specialty			0			0			0			0			0	0	0	0
Pulmonology	Medicare Specialty			0			0			0			0		9	9	0	9	9
Rheumatology	Medicare Specialty			0			0			0			0			0	0	0	0
Ophthalmology	Medicare Specialty			0			0			0		16	16		6	6	0	22	22
Radiology, Nuclear Medicine	Medicare Specialty			0			0			0			0			0	0	0	0
Radiology, Radiation Oncology	Medicare Specialty			0			0		6	6			0			0	0	6	6

UC = University of California, San Francisco; VA = San Francisco VA; C = Combined; NUCC = National Uniform Claim Committee.

## Discussion

We sought to evaluate the feasibility of executing a single SQL query on parallel OMOP instances from two separate healthcare systems. We found that the queries executed successfully and retrieved complementary information for five patients, underscoring the value of the CDM. The fact that very similar code was successfully executed and provided complementary patient data demonstrates the value of CDMs to harmonize health data from disparate systems. However, several challenges were encountered: lack of a uniform patient identifier; use of different standardized vocabularies (e.g., CPT versus ICD-10 versus SNOMED-CT procedure codes, Medicare versus NUCC provider classification, RxNORM versus National Drug Codes, HCPCS versus LOINC codes for durable medical equipment); and varied ETL mapping (e.g., to measurement versus observation tables) for the same data elements, which may not have followed CDM-specified conventions for mapping standardization across vocabularies.

The largest challenge was simply linking patients across the two healthcare systems. Because the two systems generated separate OMOP patient IDs, we had to rely on source data to link patients. Even within the source data, there was no uniform identifier other than SSN. Therefore, we had to (1) use SSNs to link patients in the source records and (2) create a crosswalk to link those SSNs with different OMOP patient identifiers in the two healthcare systems. The use of disparate patient identifiers across healthcare systems is emblematic of a significant weakness within the US healthcare system—lack of a Uniform Personal Identifier (UPI). In the past, development of a UPI has been banned due to privacy concerns, but paradoxically, using SSNs as the only unique health identifier risks even greater privacy threats because of its use in so many other domains. However, the recent growth and expansion of Qualified Health Information Networks may render the FHIR patient.identifier a de facto UPI in the US and beyond. 2 18 In 2007 the RAND Corporation estimated that the US would save nearly $77 billion per year with implementation of a UPI for 90% of patients. 19

Multiple Northern European countries have developed identification numbers that act as UPIs. Denmark uses the Danish Civil Registration System (DCRS) to generate the Central Person Register (CPR), a 10-digit number that includes a 6-digit date of birth plus a 4-digit serial number with a check digit (females even, males odd) for sex at birth. 20 Another well-implemented UPI is the Swedish Personal Identity Number (PIN). 21 Both the Swedish PIN and Danish CPR numbers are less error prone than the SSN because they incorporate a check digit. 20 21 However, their use is not limited to healthcare. Like the SSN, they are used in multiple national registries that include both healthcare and non-healthcare domains.

Another issue was use of different vocabularies and taxonomies in the source data. In their 2022 recommendations for achieving interoperable and shareable medical data in the US, Szarfman et al said the lack of comprehensive, centrally coordinated medical data collection and transmission standards results in loss of information, inefficient operations, and huge costs. 22 Our findings underscore these conclusions. During the original design of our SQL query, multiple CONCEPT_IDs for measurements (laboratories), visit occurrences, and procedures were specified based on queries of the VA OMOP instance using text matching wildcard statements. These statements generated the list of CONCEPT_IDs used to query the UCSF OMOP instance ( Table 1 ). However, no visit occurrences were identified at the UCSF using these CONCEPT_IDs, and visits were found only after adding physician specialty concepts from a different vocabulary. In addition, counts of measurements, procedures, and visits at the UCSF were much lower with these specific CONCEPT_IDs than when wildcard statements were used. This suggests that the two healthcare systems used different vocabularies and mapping.

The third challenge was variance in the ETL applied to transform source data into the OMOP CDM. Although redundancy is a strength of the OMOP CDM, it presents challenges when the same healthcare event can be represented in multiple ways. For example, one data transformation may map a laboratory procedure to CONCEPT_IDs for LOINC codes whereas another may map the same procedure to CONCEPT_IDs for CPT codes. A similar issue was encountered in a previous study that attempted to obtain medication exposure data 22 from OMOP-transformed data. In a VHA healthcare system, medication exposure was derived from dispensing events, whereas exposure was derived from physician orders in Partner's Health. Again, these challenges underscore the conclusions of Szarfman et al, who stated, “mapping and remapping from the irregular codes of each health information system to the standardized versions needed for exchanging data is an error-prone, inefficient, and costly process.” 21 Greater attention to standardized mapping could potentially address some of these issues. 23 For example, mapping a CPT code to a CONCEPT_ID could automatically populate the LOINC code CONCEPT_ID for that same procedure, and mapping a NUCC code to a non-standard CONCEPT-ID could automatically populate the corresponding (Medicare) standard CONCEPT_ID for that provider type.

Our study was limited by testing between only two institutions, for only five patients, and querying a limited number of OMOP tables (five total tables queried). In addition, we were unable to perform chart review validation of the queries as the authors do not have EHR access at both the healthcare systems. The small sample size, lack of chart review validation, and number of queried tables may have limited demonstration of interoperability, though the presence of instances of laboratory testing suggest against this being the case. Despite these limitations, results of this study may be generalizable given the standardized nature of the OMOP CDM and prior studies that note OMOP to operate similarly with other CDMs during comparison. 24 25

We are not aware of any previous publication describing experience executing a single SQL query on CDM-transformed data from two healthcare systems. This article documents the unexpected challenges we encountered when attempting to execute what should have been an easy process between two healthcare organizations with OMOP-transformed EHR data. In conclusion, we verified that use of a single SQL script to query OMOP-transformed data yielded complementary information from two separate institutions with alteration of only the OMOP patient ID and table prefixes. However, interoperability was limited by lack of a UPI, use of separate vocabularies in the source systems, and different ETL mapping. These findings suggest that a UHI, and greater attention to mapping standardization across CDM vocabularies, could further enhance interoperability.

## Clinical Relevance Statement

This paper provides an example of how a CDM might be used to obtain complementary information about patients who receive healthcare from more than one source and identifies challenges for healthcare organizations and policy makers to consider when implementing a healthcare CDM.

## Multiple Choice Questions

1. What is the purpose of the Observational Medical Outcomes Partnership (OMOP) common data model (CDM)?
  - To facilitate interoperability between different healthcare organization
  - To exchange information about clinical events from two different databases in a single language
  - To translate medical records from French to English
  - All of the above
  - Both a and b
  The correct answer is option e.
2. Which of the following are considered standardized vocabularies?
  - Systematized Medical Nomenclature of Medicine–Clinical Terminology (SNOMED-CT)
  - International Classification of Diseases 10 (ICD-10)
  - Logical Observation Identifiers Names and Codes (LOINC)
  - All of the above
  The correct answer is option e.
